# Somatic Embryogenesis Induction in Woody Species: The Future After OMICs Data Assessment

**DOI:** 10.3389/fpls.2019.00240

**Published:** 2019-03-28

**Authors:** Maria Salomé Pais

**Affiliations:** Academy of Sciences of Lisbon, Lisbon, Portugal

**Keywords:** somatic embryogenesis, woody species, cell fate reprogramming, OMICs, embryogenic competence

## Abstract

Very early somatic embryogenesis has been recognized as a powerful method to propagate plants *in vitro*. For some woody species and in particular for some coniferous trees, somatic embryogenesis induction has become a routine procedure. For the majority, the application of this technology presents yet many limitations especially due to the genotype, the induction conditions, the number of embryos produced, maturation, and conversion, among other factors that compromise the systematic use of somatic embryogenesis for commercial purposes especially of woody species and forest trees in particular. The advancements obtained on somatic embryogenesis in Arabidopsis and the development of OMIC technologies allowed the characterization of genes and the corresponding proteins that are conserved in woody species. This knowledge will help in understanding the molecular mechanisms underlying the complex regulatory networks that control somatic embryogenesis in woody plants. In this revision, we report on developments of OMICs (genomics, transcriptomics, metabolomics, and proteomics) applied to somatic embryogenesis induction and its contribution for understanding the change of fate giving rise to the expression of somatic embryogenesis competence.

## Introduction

Prediction of plant cell totipotency, the base concept of somatic embryogenesis, is due to Schleiden (1838), the author of Schleiden theory, and Schwann (1839) who postulated the capacity of individual cells from a plant to divide independently.

Later on, Haberlandt (1902) considered that plant vegetative cells could be induced to form embryos capable of giving rise to entire plants. Only around half a century after, Steward et al. (1958) and Reinert, in the same year, described the production of somatic embryos from cultured cells of *Daucus carota* (Reinert, 1958).

This achievement was the starting point for the use of a myriad of species and explant types to induce the formation of somatic embryos. As common factors, the submission of the explants to a biotic or abiotic stress and the use of the synthetic auxin 2,4-D (dichlorophenoxy acetic acid) in the culture medium constitute routine procedures recognized as inducers of totipotency leading to somatic embryogenesis, whenever the appropriate culture conditions are found.

Recognizing the great potential of somatic embryogenesis, a wide range of species have been studied ranging from monocots to dicots, and among them, herbaceous and woody/forestry species have been intensively studied (Bonga, 1995).

Attempting to succeed the production of somatic embryos from a wide range of species, factors such as age and type of explants, type of stress, auxin type and concentration and components of the induction medium have been tried (for a review see Dantu and Tomar, 2010).

While research was progressing in carrot, a number of researchers tried to find reproducible protocols to obtain and convert somatic embryos from a variety of species. As a few examples, the following can be referred for herbaceous species: *Ranunculus sceleratus* (Konar et al., 1972) and *Medicago truncatula* (Imin et al., 2005); *Zea mays* (Emons and Kieft, 1991); *Arabidopsis thaliana* (Gaj, 2001, 2004; Gaj et al., 2005), Mordhorst et al. (1997).

Somatic embryogenesis has been recognized as an ideal approach for clonal mass propagation, genetic improvement, especially of woody species that present a very long life cycle. Somatic embryogenesis provides a very useful tool for cryo-storage of useful germplasms. These advantages led to intensive research trying to establish reproducible protocols for efficient production and maturation of somatic embryos.

From the amount of woody species studied, the following species can be referred as examples: Citrus sp. (Shamouti orange) (Kochba et al., 1977); *Pinus taeda* (Becwar et al., 1990); conifers (Bonga, 1995), *Camellia sinensis* (Nakamura, 1988; Mukhopadhyay et al., 2015); Coffea sp. (Nakamura et al., 1994), *Camellia japonica* (Barciela and Vieitez, 1993); (Pedroso and Pais, 1992, 1993, 1995); *Laurus nobilis* (Canhoto et al., 1999); Chestnut (Corredoira et al., 2006, 2006a); *Picea abies* (Chalupa, 1985; Hakman et al., 1985); *Picea glauca (Park et al., 1993); Theobroma cacao* (Alemanno et al., 2008); *Cyphomandra betacea* (Correia et al., 2011); *Quercus suber* (El Maâtaoui et al., 1990; Bueno et al., 1992); *Quercus robur* (Corredoira et al., 2006); Pinus species (Häggman et al., 1999; Montalbán et al., 2015); *Alnus glutinosa* (Corredoira et al., 2013); *Fraxinus mandshurica* (Kong et al., 2012); *Larix kaempferi* (Kim, 2015), *P. abies* (Petrek et al., 2015), woody plants in general (Yuan et al., 2016), among many others.

In spite of the efforts to achieve adequate, highly reproducible protocols for somatic embryogenesis induction and embryo conversion, there is a very long way to go until this process can be used for commercial applications of important woody species. A good example is of Eucalyptus species, a forestry genus economically very important in the world industry. Since 1986, a number of Eucalyptus species (*E. grandis*, *E. citriodora*, *E. gunnii*, *E. dunnii*, *E. nitens*, *E. tereticornis*, *E. camaldulensis*, *E. glogulus*, and *E. saligna*) have been assayed using different types and age of explants, different physiological conditions of the donor plant, different types and combinations of auxins, and different environmental culture conditions. For most species, the results achieved are far from being successful. However, Termignoni et al. (1996) succeed to obtain somatic embryos presenting a high plant regeneration frequency in *E. dunnii*, this method having been subject of property rights under the Patent No. PI 9801485-4 INPI). Corredoira et al. (2015) have also reported on the production of somatic embryos of *Eucalyptus globulus* and *E. saligna* × *Eucalyptus maidenii*. For a review on somatic embryogenesis in woody plants, see Guan et al. (2016), Ochoa-Alejo (2016) and Yuan et al. (2016).

According to Fehér (2015), the mechanisms underlying auxins- and stress-induced somatic embryogenesis are not known, but both these conditions induce biosynthesis of endogenous auxins. The synthesis of endogenous auxins is considered as a crucial early step in the switch on to totipotent growth. Despite the research developed to understand the cascade of events underlying somatic embryogenesis induction and conversion, these processes are far from being understood.

Taking into account that in somatic embryogenesis, the mechanisms responsible for expression of embryogenic competence, in particular the molecular mechanisms of cell fate transition, are of crucial importance for success of somatic embryogenesis, in this paper, we provide a set of information on recent omics data regarding this embryogenesis step in an attempt to contribute to an increased knowledge on the somatic embryogenesis induction process which may be useful for future application of somatic embryogenesis as a real opportunity for mass *in vitro* propagation, germplasm conservation, genetic improvement, and breeding of woody species.

## Factors Controlling Somatic Embryogenesis

Attempts to produce somatic embryos from woody species started with the pioneer research of Rao (1965) that succeed to obtain embryo-like structures from *Santalum album*, and of Durzan and Steward (1968) and Chalupa and Durzan (1973) that reported on cell and tissue culture of white spruce (*Picea glauca*) and jack pine (*Pinus banksiana*). However, these embryo-like structures were unable to produce entire plants.

Along with time, much knowledge has been obtained on media composition, physical conditions of the culture, type of stress induction and auxin type but changing the genotype, very frequently the results expected were not reached. Also the type of donor explant is very different among the species under study. To identify possible markers of somatic embryogenesis, Pedroso and Pais (1992, 1993, 1995), working with *C. japonica*, reported on fluctuations in the levels of inorganic elements namely Ca, K, Na, Mg, S, P, and Fe and considered that higher levels of these elements may be related to embryogenic/morphogenic competence. Considering the role of these elements in living cells, the results reported for *C. japonica* account for the involvement of Ca^2+^ in phosphorylation and dephosphorylation events, and modulation of enzyme actions especially of kinases. Ca^2+^ and phosphorylation are intimately connected with homeostasis, rapid recognition of signals and with changes in biosynthetic pathways. Recently, Rivas-Sendra et al. (2017) observed a tremendous increase in Ca^2+^ concentration in cytosol and mostly in vacuoles of rapeseed embryogenic microspores compared to the levels of non-embryogenic ones. The accumulation of Ca^2+^ in the vacuole may constitute a mechanism of control of calcium homeostasis in spores under somatic embryogenesis induction conditions. These authors also verify that the concentration of Ca^2+^ in the vacuoles is inverse to the presence of callose layer, which may account for the role of this layer in inhibiting the Ca^2+^ influx into the vacuole of the embryogenic cells. Ca^2+^ has been considered to function as a second messenger. Ca^2+^ signal results from signaling fluxes of Ca^2+^ across membranes, resulting in increase/decrease of cytosolic Ca^2+^ calcium concentrations (Steinhorst and Kudla, 2013).

It is also known that morphogenesis only occurs if high Ca^2+^ levels are sustained and that DNA translation is dependent on Ca^2+^ message. Ca^2+^ through calmodulin or calmodulin-like proteins switchs/modulates enzyme actions, especially kinases. Calmodulins are encoded by a multigene. They have been considered essential for somatic embryogenesis induction from undifferentiated cells in carrot and other plants (Overvoorde and Grimes, 1994). The increased Ca^2+^ levels in embryogenesis competent cells account for its role in callose formation, in cell wall stabilization, and in mitosis after cell fate change.

The increased levels of Na^+^ and K^+^ in competent cells following the stress induction account for its role in osmotic control; electrolytic balance; stability of polyelectrolytes, of DNA, and of cell membranes; cell/cell communication and chemical uptake of organic metabolites. Phosphate is known to be involved in the synthesis of DNA, RNA, proteins, polysaccharides, and phospholipids. During somatic embryogenesis induction, differential gene expression results in synthesis of new mRNAs and proteins (Joshi and Kumar, 2013). Metabolic energy is constantly required in the biosynthesis of different metabolites prior to cell division, in cell division, and in cell wall stabilization. In morphogenesis, phosphate metabolism may become determinant for cells to become embryogenesis determined. In living cells, Mg^2+^ is required in all steps of transcription and translation through the involvement of nucleotide triphosphates and stabilizes DNA bending. Mg^2+^ forms complexes with GTP and is involved in protein synthesis and acts in tubulin polymerization. It is important to notice that S levels increase since the first hours of SE induction. In living cells, S can polymerize to –S-S and Sn units that are essential for the stability of –SH groups and of extracellular proteins. It participates in the formation of biotin and methionine and plays an important role in control of homeostasis. The increased levels of those elements after induction treatment and its flux from the non-embryogenic midrib of the leaf to the embryogenic margin account for the role of these elements in creating the conditions for somatic cells to undergo the first events to change cell fate and to start the embryogenic process. The evaluation of elements’ concentration in the explants may constitute an initial approach to determine the best explant to be used for induction.

Cell wall changes, in particular deposition of callose and cutin layers around competent cells, have been reported and considered as *in vivo* markers of embryogenic competence.

In cork oak, Rodríguez-Sanz et al. (2014a,b) suggested that cell wall remodeling by pectin esterification plays a role in somatic embryogenesis induction from immature zygotic embryos in woody species.

Studies on *Brassica napus* somatic embryogenesis have also demonstrated the presence of a layer external to the cell walls of embryogenic cells and not around the non-embryogenic ones (Namasivayam et al., 2006). These authors suggested that this layer is composed of polysaccharide/mucilage components. Similar results were described for *Coffea arabica* (Sondahl et al., 1979), Citrus sp. (Chapman et al., 2000a,b,c), *Pinus nigra* (Jasik et al., 1995) and coconut (Saenz-Carbonell et al., 2012).

Namasivayam et al. (2006) and Namasivayam (2007) considering the time and location of these layers corroborate the previous results of Pedroso and Pais (1995) according which the occurrence of this layer, also called supraembryonic network by Chapman et al. (2000a,b,c), may constitute a cellular marker of embryogenic competency.

A scanning electron microscopy (SEM) study during the induction of somatic embryogenesis from cell suspension cultures of *Phoenix dactylifera* L. performed by El-ghayaty et al. (2014) showed differences in elements accumulation during the different somatic embryogenesis stages and also considered that these features can be used as signal markers of totipotency. Parra-Vega et al. (2015) have produced an interesting demonstration on the presence of a callose layer in the embryogenic competent microspores of *B. napus*.

Interestingly Fortes et al. (2002) have demonstrated the deposition of callose and cutin layers surrounding the cells competent for morphogenesis induction, (organogenic nodule formation) in hops. Taking together the reviewed results the authors assumed that deposition of a callose and cutin layer around the cell (s) competent to undergo a morphogenic process (somatic or gametic embryogenesis or organogenic nodule formation) can be considered a marker of somatic embryogenesis/morphogenesis. As suggested by Fortes et al. (2002), the cutin layer appearing underneath the callose layer may be responsible for creating a specific cellular environment, resulting from changes in permeability and in receptors, capable of providing the conditions for the competent cells to enter the cell cycle and undergo the morphogenic process (somatic/gametic embryogenesis or organogenic nodule formation).

## Role of Omics (Transcriptome, Proteome, and Metabolome) in the Optimization and Improvement of Somatic Embryogenesis Induction in Woody Species

In spite of the very high input of data published until now, in woody species and in particular in conifers that represent a very high percentage of economically important forestry species, the molecular events underlying somatic embryogenesis induction and embryo development have been hindered by their large genome, slow growth, and long generation time.

The capacity of an explant to produce somatic embryos depends on the genotype and on many other different factors including phytohormones, proteins, and transcription factors. Different chemical compounds also act in gene expression as signals. Cross-linking between phytohormones, proteins, and transcription factors is likely to play an important role in somatic embryogenesis induction.

### Learning From *A. thaliana* Somatic Embryogenesis

Among the major bottlenecks still existing in reaching the high promises of somatic embryogenesis as a clonal propagation system of plant species in general and of woody species in particular, the recalcitrance to somatic embryogenesis induction, the low yield of somatic embryo production, and difficulties in the subsequent developmental steps of somatic embryos, in particular embryo conversion to plants, have to be mentioned. Studies have been performed in several species including model species such as *D. carota* and *A. thaliana* but their application in improvement of woody species in general and of forest trees, in particular, are far from being achieved, especially when commercial purposes are concerned.

Following the genome sequencing of *A. thaliana*, this species has soon become a model plant for cell biology and molecular studies aiming at understanding plant development, mechanisms of signaling, and resistance/tolerance to biotic/abiotic stresses, plant development, and gene regulatory networks. More recently, it provides an excellent system for studies of functional genomics, systems biology, synthetic biology, and embryogenesis *in vitro* among others. For a review, see Provart et al. (2016) and Wickramasuriya and Dunwell (2015).

The possibility to induce somatic embryogenesis in *A. thaliana*, (Huang and Yeoman, 1984; Pillon et al., 1996) has highly contributed to the identification of genes that positively regulate or inhibit SE induction, embryo maturation, and conversion. However, a drawback of using *A. thaliana* as a model species for studying expression of totipotency and somatic embryos production is the frequency of abnormal phenotypes of the somatic embryos obtained. According to Hand et al. (2016), although gametic and somatic embryos differ in origin, they share many similarities, namely the stress used to induce embryogenesis and the developmental changes that result in the formation of embryos. Comparing the results obtained for these two types of embryos, the authors have made progress in the understanding of the complex networks underlying the somatic embryogenesis process.

### Genes and Somatic Embryogenesis Induction—Transition From the Vegetative to the Embryogenic State

A number of genes differentially expressed during somatic embryogenesis induction have been isolated, which provides clues for knowledge on the synthesis of new proteins involved in somatic embryogenesis process.

The research performed in zygotic and somatic embryogenesis of *A. thaliana* allowed the authors to identify genes responsible for SE induction. According to Tsuwamoto et al. (2010), among genes putatively related with competence for somatic embryogenesis, the following can be considered: LEAFY COTYLEDON genes, LEC1 (Lotan et al., 1998) and LEC2 (Stone et al., 2008), WUSCHEL (WUS) (Zuo et al., 2002), BABY BOOM (BBM) (Boutilier et al., 2002), AGAMOUS-LIKE-15 (AGL15) (Harding et al., 2003), and AINTEGUMENTA-LIKE 5/PLETHORA 5/EMBRYOMAKER (AIL5/PLT5/EMK) (Tsuwamoto et al., 2010). These genes encode transcription factors and when overexpressed, somatic embryogenesis is promoted. Horstman et al. (2017a,b) suggested that BBM, LEC1 and LEC2 are key regulators of plant cell totipotency. According to the same authors, BBM transcription factor induces somatic embryogenesis through activation of LEC1-ABI3-FUS3-LEC2 network. In *Larix decidua*, Rupps et al. (2016) have demonstrated that LdLEC1 and LdWOX2 are mainly expressed in early embryogenesis. Recently, Lu et al. (2017) have reported on a higher expression of CASER, CmLEC1, CAMUS, and CmAGL15 genes at the initial stage of embryo formation which accounts for the role of these genes in induction of somatic embryogenesis in *Castanea mollissima*. These results combined with those of Rupps et al. (2016) open important new windows for the establishment of successful protocols for mass regeneration and improvement of economically important woody species.

Boutilier et al. (2002) have reported on the role of BABYBOOM (BBM) and AIL5 transcription factor in cell proliferation during somatic embryogenesis of Arabidopsis and Brassica. According to Tsuwamoto et al. (2010), Embryomaker gene encoding an AP2 domain transcription factor plays a crucial role in the transition from vegetative to embryogenic state. Hecht et al. (2001) have studied the expression of DcSERK (Somatic embryogenesis receptor kinase) in embryogenesis competent cells of *D. carota* and considered this gene as a marker of competence for somatic embryogenesis. According to Salaj et al. (2008), in *A. thaliana*, SERK expression precedes and coincides with early embryogenesis and again these authors considered AtSERK1 as a marker of competence for embryogenesis. Different authors have recognized SERK1 as a positive regulator of somatic embryogenesis in dicots (De Oliveira Santos et al., 2005; Ma et al., 2012; Zhai et al., 2015), monocots (Hu et al., 2005), and conifers (Li et al., 2014; Rupps et al., 2016 and Li et al., 2017a,b). Magnani et al. (2017), performing the transcripts profiling of embryogenic tissues concluded that: embryogenic cells differ from callus cells by repressing root meristem genes and genes related to biochemical pathways, while switching on transcriptional networks involved in shoot patterning, cellular re-organization; and polarized cell growth.

Recently, the regulatory roles of miR156-SPL in citrus embryogenic callus have been postulated by Long et al. (2018). According to these authors, the somatic embryogenesis competence of wild kumquat (*Fortunella hindsii*) was significantly enhanced by over-expression of csi-miR156a. Among the biological processes the authors suggested to occur in response to miR156 over-expression, hormone signaling pathways, stress responses, DNA methylation, and the cell cycle have to be mentioned. Wu et al. (2015) succeeded in profiling a set of miRNAs/siRNA and their targets in *Citrus sinensis* somatic embryogenesis system. By comparing the expression levels between embryogenic and non-embryogenic cells/tissues, the authors showed that the miRNA/siRNA-mediated differential regulation of specific biological processes and TFs may be associated with competence for somatic embryogenesis. Taking into account the great amount of miRNA/siRNA, and comparing its expression in non-embryogenic and embryogenic callus, the authors suggested the involvement of miRNAs and SiRNAs in somatic embryogenesis potential in citrus, by down-regulation of most of miRNAs and siRNAs accompanied by up-regulation of a few conserved miRNAs in the embryogenic tissues. The same authors have postulated that these conserved miRNAs should derepress important development-required and stress-response genes, at the same time that some TFs should be suppressed to finetune the embryogenic potential.

Szyrajew et al. (2017) have demonstrated the role of miRNA-controlled regulatory pathways in Arabidopsis somatic embryogenesis induction. These authors suggested that this microRNA might control fate transition by the regulation of TRANSPORT INHIBITOR-1 (TIR1) and AUXIN F-BOX PROTEIN (AFB), both of them auxin receptor genes.

The same authors reported on the identification of a set of candidate miRNAs that may play an important role in the regulatory network controlling the transition from vegetative to embryogenic state. The transcription factor miR393 may also play a role in the control of Arabidopsis somatic embryogenesis induction (Wójcik and Gaj, 2016). These findings contribute to understand the role of auxin in the somatic embryogenesis induction medium as a key regulatory of the pathway underlying cell fate transition.

This knowledge will be helpful for identifying genes targeted by the candidate miRNAs in SE induction and its application to woody species.

In fact, SE induction depends from hormone signaling, responses to different stresses, the genotype under study, and DNA methylation status in order for the competent cells to enter the cell cycle.

Yue et al. (2016) have presented a very good demonstration of a hormone crosstalk network that may play a very important role in developmental processes following different stress signals in Arabidopsis. According to Ascenzi-Fabado et al. (2017), changes of chromatin features, in particular histone modifications, occur after abiotic stress treatments. Many of these changes are associated with genes that are transcriptionally regulated by the stress applied to the explant.

These findings suggest the existance of an integrative regulation of somatic embryogenesis in woody species economically important in particular in Citrus species improvement.

Combining the molecular and biochemical knowledge on transcriptional regulation under stress conditions will help in the generation of predictive network models that may regulate change of fate in somatic embryogenesis induction in plants.

### Somatic Cells Dedifferentiation and Auxins

A condition for somatic embryogenesis induction is the presence of an auxin in the induction medium. Iwase et al. (2011a,b) have demonstrated the role of the transcription factor Wind1 in somatic cells dedifferentiation in the absence of exogenous auxin and/or cytokinin. According to Nowak et al. (2015), LEC2 is a key regulator of somatic embryogenesis by stimulation of auxin synthesis. It is possibly related to ERF022. These authors considered that auxin-ethylene interactions are putatively controlled by ERF022 and LEC2 and their targets. De la Peña et al. (2015) reported on changes in DNA methylation patterns that are associated with regulation of BBM and LEC genes among others. This correlation among BBM and LEC genes with DNA methylation may account for a role in crosstalk among gene regulation and auxin effect, essential for change of vegetative state to embryogenesis competence induction. Nonetheless, Klimaszewska et al. (2009) working on somatic embryogenesis of *Pinus pinaster* (three embryogenic culture types) reported that no significant differences in DNA methylation levels are observed when embryogenic and non-embryogenic lines are compared. The methylation values varied from hypomethylation in embryogenic lines to hyper-methylation in non-embryogenic ones. These results on DNA methylation values account for the need of more research on this subject in conifers as well as on its relationship with age of the donor material.

Later on, Klimaszewska et al. (2011) succeeded to identify CHAP3A, VP1, WOX2, and SAP2C that appear to be exclusively expressed in the early stages of somatic embryogenesis, which may account for an important role of these genes in the very early steps of somatic embryogenesis and should potentially be used as markers of embryogenesis.

Wójcikowska and Małgorzata (2017) reported on significant increase of the auxin regulation factors ARF5, ARF6, ARF8, ARF10, ARF16, and ARF17, ARF5 being the most auxin responsive. Aintegumenta-like (AIL) has also previously been shown to promote somatic embryogenesis in the absence of external growth regulators. Horstman et al. (2014) emphasized the relationship of ALE genes with auxins and their involvement in gene regulatory networks. AIL genes regulate somatic embryogenesis, meristem development, organ initiation, and growth and interact with auxin pathways along plant development and their function is dosage dependent, Horstman et al. (2014).

In a very interesting review, Horstman et al. (2017a,b) presented data accounting for the role of transcription factors in chromatin-modifying proteins. These authors have elaborated a very interesting model of the molecular regulation of somatic embryogenesis in Arabidopsis, emphasizing the regulation among transcription factors and expression of genes involved in auxin and cytokinin pathways. The levels of phytohormones (in particular auxins, cytokinins, abscisic acid, jasmonates, and salicylic acid) are different throughout *P. abies* somatic embryogenesis (Vondrakova et al., 2018). These authors demonstrate the role of each of the phytohormones in specific embryogenic steps, assuming that clear correlations may exist among the different profiles of endogenous phytohormones and specific somatic embryogenesis stage (since induction until maturation). Cao et al. (2017) reported on a crosstalk among auxin and ethylene in the initiation of cotton somatic embryogenesis. According to these authors, changes in endogenous auxin levels may be one of the first events responsible for SE induction. They also suggested a role of LRR-RLKs in the SE process and in the regulation of the somatic embryos differentiation rate. Gliwicka et al. (2013) have previously studied the transcription factors and transcriptome associated with Arabidopsis somatic embryogenesis induction and demonstrated the combined effects of stress and hormone signaling following the stress imposed by *in vitro* culture. The same authors considered that among the transcription factors showing somatic embryogenesis-specific expression are those involved in stress and hormone responses. Working with *T. cacao*, Florez et al. (2015) have enhanced somatic embryogenesis by using the homologous BBM transcription factor.

Liu et al. (2018) have demonstrated that the auxin-inducible WUSCHEL-RELATED HOMEOBOX 11 (WOX11) activates LBD16 expression and promotes pluripotency acquisition. LBD16 is only expressed in callus cells growing on callus induction medium, being inhibited when calli are transferred to shoot induction medium which accounts for the role of LBD16 in the acquisition of pluripotency. Liu et al. (2014) and Sheng et al. (2017) have pointed out that the WOX11-LBD16 pathway is essential for adventitious root formation from Arabidopsis leaf explants and suggested that the acquisition of pluripotency requires the expression of genes related to root primordia identity.

Different authors have pointed out that AtLEC1 acts as a central regulator of cell fate change and participates in different signaling pathways such as hormone and light signaling pathways either in somatic or in zygotic embryogenesis (Junker et al., 2012; Huang et al., 2015). AtLEC2 is considered as a regulator of embryogenesis by repressing GIBBERELLIN 3-BETA-DIOXYGENASE 2 (AtGA3ox2) and promoting the auxin pathway through up-regulation of the auxin biosynthesis genes AtYUCCA2 and AtYUCCA4, and of the auxin signaling gene INDOLE-3-ACETIC ACID INDUCIBLE 30 (AtIAA30) (Curaba et al., 2004; Braybrook et al., 2006; Stone et al., 2008).

According to Yang et al. (2012), auxin-related transcripts belonging to IAA biosynthesis, indole-3-butyric acid (IBA) metabolism, IAA conjugate metabolism, auxin transport, auxin-responsive protein/indoleacetic acid-induced protein (Aux/IAA), auxin response factor (ARF), small auxin-up RNA (SAUR), Aux/IAA degradation, and other auxin-related proteins, make part of a complex system of auxins that play a role in achieving a set of purposes during somatic embryogenesis. Jin et al. (2014) have also reported on the expression of auxin-responsive genes, tryptophan synthase beta-subunit 2 (TSB2), IAA amido synthetase (GH3), Aux/IAAs, and auxin response factors in the sequence of somatic embryogenesis induction in cotton (*Gossypium hirsutum*).

Wang et al. (2018) have demonstrated that over-expression of GhSPL10, a target of GhmiR157a, increases free auxin and ethylene content. According to the same authors, expression of associated signaling pathways activates flavonoid biosynthesis and promotes initial cellular dedifferentiation and callus proliferation. Silencing of the flavonoid synthesis gene F3H blocks callus formation, while exogenous application of different types of flavonoids promote callus proliferation, at the same time that cell cycle-related gene expression occurs. Inhibition of ethylene synthesis severely inhibits callus initiation. On the contrary, activation of ETHYLENE SIGNALLING genes through 1-aminocyclopropane 1-carboxylic acid treatments or inhibition of the ethylene negative regulator CTR1 by RNA interference promotes EIN2 over expression, flavonoid-related genes expression, and flavonol accumulation. These results show that up-regulation of ethylene signaling and activation of flavonoid biosynthesis are associated with cell dedifferentiation and callus proliferation, accounting for an important role of the GhmiR157a–GhSPL10 gene module in somatic embryogenesis regulation.

The results reported by different authors, in particular by Tao et al. (2016), Trontin et al. (2016), and Wang et al. (2018), account for an important role of auxins in SE induction through the control of genes like LEC, WUS, FUS, SPL and a set of transcription factors. Tian et al. (2017), reviewing the results on the novo shoot regeneration, pointed out that when explants are cultured on auxin-rich medium, a group of PLETHORA (PLT) transcription factors are required for pluripotency acquisition, and afterward the ARABIDOPSIS RESPONSE REGULATOR (ARR)-mediated cytokinin pathway, together with class III homeodomain leucine zipper (HD-ZIP III) transcription factors, directly activates WUSCHEL (WUS) expression for reprogramming the shoot’s fate when cultured on a cytokinin-rich medium. Xu et al. (2018) have demonstrated that the auxin-induced LATERAL ORGAN BOUNDARIES DOMAIN (LBD 29) functions as trigger of cell reprogramming in callus formation. According to the same authors, LBD 29 targets hundreds of genes, being among them genes related to methylation, reactive oxygen species (ROS) metabolism, cell wall hydrolysis, and lipid metabolism that are quickly activated after auxin treatment. According to Sugimoto et al. (2018), the prime characteristic of plant regeneration is cell fate reprogramming induced by wounding, stress, or hormones.

Wu et al. (2017) pointed out that auxins play a crucial role in the coordination of morphogenesis and development of plant reproductive organs, particularly in the signal-transduction cascade that leads to the reprogramming of gene-expression patterns previous to embryo formation. These authors have recently reported on the involvement of strigolactones in the tomato embryogenic process through crosstalk with other hormones, mostly auxins.

### Somatic Embryogenesis as a Stress-Induced Process

Dedifferentiation, a requisite for somatic embryogenesis to occur, depends on the stress conditions applied.

One of the conditions for induction of somatic embryogenesis either in woody or in herbaceous plants is the use of a stress (temperature, wounding, heavy metal ions, starvation, osmotic stress, wounding among many others). As a matter of fact, the same type of stress can induce SE in different species. This fact brings to mind the idea that a stress, whatever it is, may be responsible for switching on an initial reaction that may induce dedifferentiation of the embryogenically competent cells. The capacity to display competence for embryogenesis is very different among the plant species. The stress imposed for somatic embryogenesis induction triggers signals that play an important role in cell reprogramming.

As a reaction to stress, plants may enter dedifferentiation, a step prior to acquiring a new cell fate. According to Grafi and Barak (2014), stress induces a signaling pathway and undergoes plant cell dedifferentiation leading to acquisition of a new cell fate, and somatic embryogenesis is initiated. Efforts have been undertaken to understand the basis of cell reprogramming after stress induction. Iwase et al. (2011a,b) suggested that the AP2/ERF transcription factor WIND1 (WOUND INDUCED DEDIFFERENTIATION) controls cell dedifferentiation in Arabidopsis and that WIND1 functions as a key molecular switch for plant cell dedifferentiation. Later on, it had been demonstrated that WIND1 plays a role in the acquisition of competence for callus formation and regeneration in Arabidopsis, rapeseed, tobacco, and tomato (Iwase et al., 2013, 2015). Iwase et al. (2017, 2018) reported on the role of WIND1 in the endogenous balance between auxin and cytokinin in somatic embryogenesis-responsive explants. These authors also suggested that in activated explants a dynamic metabolic reprogramming of WIND1 takes place including the production/accumulation of different compounds namely proline, gamma aminobutyric acid (GABA), and putrescine.

Proline is known as an important molecule used by plants to combat stress. Polyamines have been involved in plant abiotic stress responses (Gill and Tuteja, 2010). Different studies have revealed the function of polyamines in stress tolerance mostly by modulating the homeostasis of reactive oxygen species (ROS). Polyamines should function directly or indirectly in the regulation of antioxidant systems or inhibition of ROS production (Liu et al., 2015). Liang et al. (2013) pointed out that proline metabolism stimulates cell-signaling pathways through increase of reactive oxygen species (ROS) formation that promote either cellular apoptosis or cell survival.

Polyamines have been suggested as crucial molecules that enhance cell proliferation and regeneration in plants (Arun et al., 2016). According to Pedroso et al. (1997), soluble and insoluble conjugated putrescine and soluble conjugated spermidine might be related to the formation and development of globular embryos. Shoeb et al. (2001), considering their results on rice somatic embryogenesis, suggested that polyamines could be used as biomarkers of rice somatic embryogenesis. Wu et al. (2009) have reported on the involvement of polyamines in somatic embryogenesis of *C. sinensis*, following induction by osmotic stress.

The molecular mechanisms underlying the accumulation of polyamines (PA) following stresses, as well as the expression of PA biosynthetic genes and its transcriptional regulation network are far from being understood. PA biosynthesis and accumulation associated to gene expression, physiological, biochemical, molecular, and genetic approaches will certainly contribute to understanding the intricate regulation of PA synthesis under stress, as well as the crosstalk between TF-mediated signaling pathways.

The resulting knowledge will surely contribute to better understand the role of polyamines in somatic embryogenesis induction.

Results from Wimalasekera et al. (2011) suggest a role of polyamines as signaling mediators, with nitric oxide being a potential mediator of polyamines action. Pedroso and Pais (personal communication) have studied the expression of the oxidative stress enzymes superoxide dismutase (MnSOD; FeSOD and Cu/ZnSOD), ascorbate peroxidase, glutathione reductase, and catalase along with direct SE induction in *C. japonica* leaves. The activity of these enzymes increased until the formation of globular structures and was higher for IBA-induced material. The authors also found a correlation between the expression of SODs and the values of Cu, Zn, Fe, and Mn at the embryogenic margin of the leaf. In fact, the results obtained account for a flux of these ions from the midrib to the leaf margin where the vegetative cells undergo a dedifferentiation process. The increase in the activity of oxidative stress enzymes may account for the protection of the embryogenic-responsive cells against the oxidative burst promoted by the stress induced with further dedifferentiation of competent cells to enter cell cycle.

In camphor, Shi et al. (2016) have reported on up-regulation of genes from the families GH3, PIN, INDOLEACETIC ACID-INDUCED PROTEIN (Aux/IAA), ARF, HSP, LATE EMBRYOGENESIS ABUNDANT (LEA), CEM6, H3-1, SERK, CALMODULIN (CAM), CALCIUM-DEPENDENT PROTEIN KINASE (CDPK), BBM, APETALA2 (AP2), and ERF after sucrose treatment. SERK genes have been identified in many SE induction processes. Ma et al. (2012) identified a new SERK gene and demonstrated its autophosphorylation activity in single competent cells and competent cell clusters of *Ananas comosus*. These authors pointed out the role of this gene in somatic embryogenesis induction. Hu et al. (2005) have previously suggested a main role of SERK1 in *Oryza sativa* SE induction. Hecht et al. (2001) considered that when the levels of AtSERK1 are increased, competence for somatic embryogenesis is promoted. Recently, Li et al. (2017a,b) demonstrate the over-expression of SERK-like genes following biotic and abiotic stresses. It is worthwhile to assume that the stress conditions imposed on the explants for SE induction may activate SERK genes coding for transmembrane SERK proteins capable of signal perception and transduction, giving rise to a cascade of events responsible for change of cell fate from competent cells. These results lead the authors to consider the expression of SERK genes as a marker of somatic embryogenesis.

Transcription factors of LEC, BBM, AP2, ERF, DREB, WUSCHEL-RELATED HOMEO-BOX (WOX) families have also been suggested as playing critical roles in camphor somatic embryogenesis process. Yingbo et al. (2017) have demonstrated that HvSERK1 genes are up-regulated in barley microspore embryogenic culture induced upon salt stress, which accounts for its role on induction of embryogenic competence of barley microspores. A similar function has been proposed by Ge et al. (2012) for SE in Valencia sweet orange somatic embryogenesis. Studies on somatic embryogenesis induction following sucrose-induced stress in camphor tree have revealed the differential expression (up-regulation) of GLUTATHIONE S-TRANSFERASES (GSTs), GERMIN-LIKE PROTEINS (GLPs), heat shock proteins (HSPs), chitinases, and β-1, 3-glucanases that may be related to the acquisition of cell embryogenic competence (Shi et al., 2016). Dalton et al. (2009) suggested a role of GSTs in providing physiological flexibility to cope with different stresses. The same authors reported on GST levels’ increase with aging, which suggests that they may play a role in plant senescence. Givaty Rapp et al. (2015) considered that senescence is a process of trans-differentiation, which means the conversion of a cell type to another. According to these authors, dedifferentiation means that any differentiated cell that keeps its genome integrity can, under specific conditions, return to a primordial state before changing its fate. Zhou et al. (2016) have pointed out that ROS homeostasis is crucial for initiation and maintenance of dedifferentiation, while mild oxidative conditions promote redifferentiation. The same authors have also assumed that somatic embryogenesis in cotton plants is modulated by the interplay between ROS and auxin homeostasis.

The stress applied for induction of SE in plants may result in chromatin decondensation and expression of a set of transcription factors encoding genes mentioned before. The deposition of a cutin layer around the embryogenic competent cells after stress induction and auxin treatment, reported in this revision, may account for the altered permeability and creation of conditions for entering senescence with consequent change of cell fate. Rodríguez-Sanz et al. (2014a,b) studying *Q. suber* microspores and immature zygotic embryos *in vitro* embryogenesis have demonstrated that phytohormones namely auxins play a role in signaling cell wall and chromatin remodeling activating specific morphogenic pathways interconnected in complex regulatory networks leading to reprogramming of competent cells for embryogenesis induction. According to these authors, DNA hypomethylation and cell wall remodeling by increasing pectin esterification are concomitant with increase in auxin levels at early stage of somatic embryo formation. Solís et al. (2012) demonstrated that in the induction of *B. napus* pollen embryogenesis, the epigenetic reprogramming is related to a decrease in DNA methylation. It has also been suggested that the microspore reprogramming to embryogenesis entails a change in the expression pattern of BnMET1a-like (METHYLTRANSFERASE-LIKE) at the transcriptional level, which highly correlates with the DNA methylation dynamics (DNA hypo-methylation and BnMET1a-like expression) (Solís et al., 2012).

According to De la Peña et al. (2015), changes in DNA methylation patterns induced by auxins are related to regulation of WUS, BBM1, and LEC genes among others. The same authors suggested that since the lowest level of DNA methylation is always found in embryogenic cells, DNA hypomethylation may be related to signaling which, at the end, leads to somatic embryogenesis induction. Alterations in DNA methylation levels and histone modifications have been reported for somatic embryogenesis induction from herbaceous and woody species, namely *Coffea canephora* (Nic-Can et al., 2013), *Castanea sativa* (Viejo et al., 2010), *Q. suber* (Rodríguez-Sanz et al., 2014a,b; Pérez et al., 2015), *Acca sellowiana* (Fraga et al., 2012; Cristofolini et al., 2014), and gymnosperms such as *P. abies* (Yakovlev et al., 2014) and *Picea omorika* (Levanic et al., 2009), *P. pinaster* (Klimaszewska et al., 2009) and *P. nigra* (Noceda et al., 2009) and Larix × eurolepis (Teyssier et al., 2014). DNA methylation and several histone lysine methylation states are traditionally considered to exert repressive effects on gene expression (Mozzetta et al., 2015; Rao et al., 2017; Schuettengruber et al., 2017). For a review, see Schvartzman et al. (2018). Guan et al. (2016) have produced an interesting model of the regulatory genes controlling somatic embryogenesis in Arabidopsis. Also, in Arabidopsis, Lee et al. (2016) have reported on the role of histone deacetylation in cellular dedifferentiation.

Ikeuchi et al. (2018) have postulated on the existence of a gene regulatory network associated with plant cell reprogramming. According to these authors, wound- and/or hormone-induced signals present intense crosstalk and regulate several common reprogramming-associated genes *via* multilayered regulatory cascades. From those genes, PLETHORA 3 (PLT3), ENHANCER OF SHOOT REGENERATION 1 (ESR1) and HEAT SHOCK FACTOR B1 (HSFB1) may function as critical factors that potentially connect upstream stimuli to downstream developmental steps. The same authors pointed out that wound-inducible APETALA 2/ETHYLENE RESPONSE FACTORs (AP2/ERFs) may function as regulators of these key genes, which, in turn, undergo feed-forward cascades controlling downstream targets related to callus formation and organ regeneration. They also reported on other regulatory pathway, regulated by LATERAL ORGAN BOUNDARY/ASYMETRIC LEAVES 2 (LOB/AS2) transcription factors that may play a different but partially overlapping role with the AP2/ERFs in the putative gene regulatory cascades. In this very interesting paper, the authors not only provide a scheme of the global reprogramming network but also detail sub-networks highlighting cytokinin/auxin/wound-mediated pathways regulating plant cell reprogramming. To the best of our knowledge, this is the first report on a gene regulatory network governing plant cell reprogramming.

Stress triggers cell reprogramming in plants (Grafi et al., 2011). Wounding was considered the most essential trigger of plant cell reprogramming (Ikeuchi et al., 2016, 2017; Radhakrishnan et al., 2018).

According to Iwase et al. (2018), four AP2/ERF transcription factors (WOUND INDUCED DEDIFFERENTIATION1): WIND1, WIND2, WIND3, and WIND4 are key wound-inducible regulators. WIND1 promotes somatic cells reprogramming and WIND1-overexpressing plants exhibit somatic embryogenesis in culture (Ikeuchi et al., 2013; Iwase et al., 2015). According to the same authors, high WIND1 expression promotes organ regeneration during *in vitro* tissue culture. Iwase et al. (2013, 2015) pointed out that the ectopic expression of At. WIND1 induces cell reprogramming in different plant species, which accounts for considering that the WIND1-induced reprogramming pathway is conserved in different plant species. In plants, phytohormones are not the only supplements that influence plant regeneration. Different low-molecular weight compounds have been added to the culture medium to induce expression of somatic embryogenesis competence, cell proliferation, and regeneration efficiency (Karami and Saidi, 2010).

The list of genes mentioned above is far from being exhaustive. However, the data shown account for the intensive research and for the knowledge acquired on cellular events taking place under the conditions commonly used for plant somatic embryogenesis induction.

In spite of the enormous amount of data gathered from transcriptomic research developed in woody species and in model plants (*D. carota* and *A. thaliana*) aiming at understanding the complex network of regulatory mechanisms governing the expression of competence for somatic embryogenesis, many gaps still have to be fulfilled before considering the possibility of using the embryogenic process as generally applicable to woody species and of its possible contribution for the improvement of somatic embryogenesis protocols required for using this system in mass propagation of woody species, in particular in conifers, to satisfy forestry needs.

Taking profit of the rapid publication of genome sequences of important woody species, including conifers, and making use of the data gathered by transcriptomic studies, it is possible to foresee advancements in the control of the precise conditions needed to successfully induce somatic embryogenesis competence in a controlled way.

Cell reprogramming, is increasingly considered a critical phenomenon in tissue regeneration, aging, and cancer (Folguera-Blasco et al., 2018). According to these authors, cellular aging and its reversal, which should correspond in plant cells to differentiation (aged cell) and dedifferentiation (young cells), may result from stochastic translation of metabolic inputs into resilient/plastic cells states. Taking into account the importance of plant metabolism in the control of cell fate, these authors conceived a computational model capable of predicting the likelihood of cell reprogramming in response to changes in aging-related metabolites. Although this model has been developed for studies on cancer development, considering the molecular relationship between cell metabolism and chromatin, we are tempted to foresee the possibility to design a similar predictive computational model of epigenetic regulation of cell fate reprogramming in plants’ somatic embryogenesis which would highly contribute to the understanding of this process and to its induction using reliable conditions.

Understanding the mechanisms regulating plant cells’ fate change and the mechanisms underlying cellular plasticity in plants could help to find new strategies to improve plant regenerative capacity, which should have a great impact on agriculture and forestry.

### Proteins and Somatic Embryogenesis Induction—Transition From the Vegetative to the Embryogenic State

Studies have been performed in order to understand the complex process of somatic embryogenesis induction. De Vries et al. (1988), working with *D. carota*, have emphasized the need of phytohormone-controlled glycosilation of extracellular proteins for somatic embryogenesis to occur. In *C. japonica*, Pedroso et al. (1995) demonstrated the presence of specific polypeptides in competent cells. Differences in the percentage (91% EC; 66% NEC) were reported. From the 43 polypeptides related to somatic embryogenesis, 2 E1 (pl 5.6; mol. wt. 43.5) and E2 (pl 6.0; mol. wt. 25.7) appeared specifically related to SE. The E2 polypeptide may correspond to an osmotin-like protein also referred by Helleboid et al. (2000) to be associated with SE induction. In *C. japonica*, the 43-kDa proteins may function as a chloroplastidial signal recognition particle pathway (UniProtKB - O22265 (SR43C_ARATH). Boyer et al. (1993) have previously emphasized the role of embryogenesis-related proteins in the induction of somatic embryogenesis in Chicorium intibus. Helleboid et al. (2000) have also identified three somatic embryogenesis-related PR proteins in Chicorium intibus somatic embryogenesis induction medium and suggest they are b-1-3-glucanases. According to those authors, these proteins may be involved in callose degradation during somatic embryogenesis.

The role of chitinases and arabinogalactan proteins as signal molecules has also been reported in *P. abies* somatic embryogenesis induction by, Egertsdotter and von Arnold (1995), and Dyachok et al. (2002). According to Van Hengel et al. (2001) and Dyachok et al. (2002), oligosaccharides released from arabinogalactan proteins by the action of a chitinase function as signal molecules that stimulate somatic embryogenesis. Cairney and Pullman (2007) have pointed out the role of arabinogalactan proteins (AGPs), chitinases, and lipochitooligosaccharides in SE induction of gymnosperms and angiosperms. AGPs and glycosylated polypeptides have been described as stimulators of somatic embryogenesis (Cairney and Pullman, 2007). Dyachok et al. (2002) suggested that an endogenous lypophilic chitin oligosaccharide (LCO) acts as a signal molecule stimulating poly-embryogenic masses and early embryo development in Norway spruce.

In spite of the huge amount of results produced to find adequate protocols for SE induction of important woody species and in particular of forest species, most improvements have resulted from empirical modifications of previously published protocols.

Along with time, enormous efforts have been made to identify proteins related to plant somatic embryogenesis induction and, in particular, in woody plants—*Q. suber* (Gomez-Garay et al., 2013), *Vitis vinifera* (Marsoni et al., 2008; Zhang et al., 2009), *C. sinensis* (Pan et al., 2009), *C. betacea* (Correia et al., 2012), *Persea americana* (Guzmán-García et al., 2013), *T. cacao* (Noah et al., 2013), *P. pinaster* (Morel et al., 2014; Niemenak et al., 2015), *Elaeis guineensis* (de Carvalho Silva et al., 2014), and Larix principis-rupprechtii (Campos et al., 2016, 2017), Zhao et al., 2015). The main research focused on expression of competence for SE and on comparison between embryogenic and non-embryogenic cells. Elhiti et al. (2013) have identified 51 proteins (for a view, see in the paper) considered as functioning in early somatic embryogenesis. These authors pointed out nine candidates that may play a critical role during the dedifferentiation phase. The putative molecular functions are DNA binding, protein binding, and UDP-forming activity. These candidate proteins were found to play roles in secondary cell wall formation, response to auxins among other functions including response to brassinosteroid, and jasmonic acid metabolisms. According to the same authors, the biological functions predicted to these proteins account for the role of the morphological and cytological separation of the embryogenic cells from the surrounding ones resulting from cell wall thickening, as reported elsewhere by Pedroso and Pais (1995) and by Fortes et al. (2002).

A role on the induction of changes in the molecular network regulating the hormonal responses in the somatic cells committed to embryogenesis as a prerequisite for a somatic cell to transit from the original developmental fate to dedifferentiated status has also been suggested.

According to Elhiti et al. (2013), these proteins may be involved in apoptosis, hormone signal transduction, GTPase signal transduction, transcription regulation, chromatin remodeling, cell cycle regulation, microtubule organization, cellulose biosynthesis, and metabolic activity. In line with the proteins identified and the corresponding genes, the authors support a role of jasmonic and salicylic acid in somatic embryogenesis induction.

Different authors reported on different functional categories of proteins. In Vitis vinífera, Marsoni et al. (2008) have demontrated the up-regulation of several stress-related proteins, namely two forms of cytosolic ascorbate peroxidase and one glutathione-S-transferase (GST). This is in agreement with data according to which different types of stresses play important roles in the switch from vegetative to the embryogenic fate. Several authors have suggested that oxidative stresses resulting from the high levels of ROS (reactive oxygen species) are responsible for somatic embryogenesis enhancement (Pasternak et al., 2002; Caliskan et al., 2004). Maraschin et al. (2005) considered that ROS might function as a second messenger in auxin stress-induced embryogenesis. In loblolly pine, it has been demonstrated that changes in the redox environment *in vitro* improve somatic embryogenesis induction and early stages of somatic embryos’ development (Pullman et al., 2015). Uncontrolled ROS production may cause oxidative burst with consequent severe cell damage. To prevent this damage, embryogenic cells require mechanisms of ROS scavenging. The identification by Marsoni et al. (2008) of two isoforms of cytosolic ascorbate peroxidase, a known free radical scavenging protein, is in agreement with the role of this enzyme in the control of ROS accumulation following stress. Steinhorst and Kudla (2013) emphasized the role of ROS and calcium in cell signaling. Other than stress-response proteins, many other have been identified and organized in functional categories, namely signal transduction, cell proliferation, and cell wall metabolism. Zhang et al. (2009) in *V. vinifera* reported on a predominant expression of acidic ascorbate peroxidase and isoflavone reductase-like proteins in embryogenic callus. In tamarillo, Campos et al. (2016, 2017), Correia et al. (2016a,b) identified metabolism-related proteins, heat-shock, and ribosomal proteins expressed exclusively or predominantly in embryogenic callus. In embryogenic callus of larch, Zhao et al. (2015) reported on up-regulation of proteins involved in developmental metabolic processes such as ADP-ribosylation factor, GTPase-activating proteins, triosephosphate isomerase as well as proliferating cell nuclear antigen and suggested that these proteins can be considered candidate markers of somatic embryogenesis in larch. Cinnamyl alcohol dehydrogenase and pathogenesis-related protein 5 have also been considered as playing a role in expression of embryonic competence (Guan et al., 2016).

Recently, the role of chromatin modifications in SE induction has been emphasized De la Peña et al. (2015). Chromatin organization induces the expression or repression of genes which depends on the degree of its compaction in a specific locus (Tamaru, 2010). This chromatin compaction is due to histone modification and DNA methylation. Solís et al. (2012) reported on a decrease in DNA methylation during the microspore somatic embryogenesis in *B. napus*. The same authors verified an increase of DNA methylation during embryo differentiation. It is possible to formulate the hypothesis that the transition from a vegetative to an embryogenic stage implies changes in the genome organization and that, as a consequence, chromatin modifications may occur. The authors have assumed that in parallel with DNA methylation, histone modifications are very important for microspore embryogenesis induction in *B. napus*. Solís et al. (2012) reinforced the role of DNA methylation and expression of MET1a-like gene in pollen reprogramming to embryogenesis. According to Rodríguez-Sanz et al. (2014a,b), heterochromatinization as well as the histones H3Ac, H4Ac, and HAT may participate in cell reprogramming events taking place during expression of competence for somatic embryogenesis while H3K9me2 and HKMT may play a role in embryo cell differentiation. The same authors working with *Q. suber* microspores and immature zygotic embryo-derived somatic embryogenesis reported on the expression pattern of BnMET1a-like genes that codify for DNA methyltransferases and on its correlation with variations in the levels of DNA methylation in the embryogenic process. These results account for a role of DNA hypomethylation in *Q. suber* somatic embryogenesis induction, an assumption that has also been formulated by El-Tantawy et al. (2014), working on *Hordeum vulgare* microspores-derived somatic embryogenesis. Changes in DNA methylation patterns are associated with the regulation of several genes involved in SE, namely WUS, BBM1, and LEC De la Peña et al. (2015).

Pérez et al. (2015) working also on somatic embryogenesis in *Q. suber* have reported on the expression pattern of genes coding for proteins related to chromatin modification and remodeling—namely, two histone deacetylases (HDACs), HDA6 and HDA19, two histone mono-ubiquitinases (HUB1 and HUB2), a histone H3 kinase (AUR3), PICKLE and VP1/ABSCISIC ACID INSENSITIVE 3-LIKE 1 (VAL1). Inhibition of histone deacetylases interferes with somatic embryogenesis induction in conifers (Uddenberg et al., 2011). According to Nic-Can et al. (2013), a decrease in DNA methylamine and reduction of repressive H3K9me2 and H3K27me3 should constitute key steps in triggering cellular dedifferentiation to achieve cell totipotency. The restore of these histones may constitute a regulatory mechanism for adequate embryo development in *C. canephora*. Regulation of LEC1 and BBM1 expression by the H3K27me3, accompanied by the repression of WOX4 by H3K9me2, is coherent with the hypothesis that epigenetic mechanisms may play a role in the control of embryo onset and development during somatic embryogenesis.

The research developed along time has demonstrated that DNA methylation is an important epigenetic mechanism—taking place in somatic embryogenesis induction. Deciphering the role of methyltransferases during somatic embryogenesis will help to understand how these enzymes participate in the set-up of morphogenic processes in plants and will contribute for the obtention of somatic embryos from woody plants.

Once somatic cells change cell fate, activation of several signal cascades leading to the acquisition of meristematic cell’s fate and entering cell divisions give rise to the first steps of somatic embryos’ formation. According to Hemerly et al. (1999), key cell cycle elements, such as CDK complexes, and the mechanisms used to regulate their activity are highly conserved. The variety of mechanisms that control CDK activity reflects the complexity of internal and environmental signals to which higher eukaryotes can respond. Liese and Romeis (2013) have pointed out CDPk function depends from calcium. These results agree with the important role of calcium in signaling and further entrance in cell division after dedifferentiation. Very early, Overvoorde and Grimes (1994) have emphasized the role of calcium and calmodulin in carrot somatic embryogenesis. Such mechanisms can be differentially modulated to provide distinct types of cycles that can be used for different roles. In plants, this network is probably even more intricate. According to Blomme et al. (2014), the main fundamental factors of the eukaryotic cell cycle are the CYCLIN-DEPENDENT KINASES (CDKs) that interact with CYCLINS (CYCs), regulatory proteins that determine the activity and substrate specificity of the CDK/CYC complexes.

Gene regulation and corresponding protein expression at this stage of embryogenesis can be grouped into two main categories: signal transduction cascade and cell cycle initiation. In plants, cell division is under the control of a complex mechanism regulated by cyclin-dependant kinesis that, following activation, controls cell cycle initiation and progress. According to cytokinin- and auxin-induced proteins (CDKA1) and (CDC2A) play crucial roles in the early phase of embryogenesis. Cell-cycle genes have been correlated with cell competence and cell reprogramming during regeneration (Ishikawa et al., 2011).

### Negative Regulators of Somatic Embryogenesis

At the same time that research is performed to unravel the factors underlying early events in somatic embryogenesis induction, efforts have been developed to understand the negative regulation of SE. Ikeuchi et al. (2015) have demonstrated that POLYCOMB REPRESSIVE COMPLEX 2 (PRC2), a chromatin regulator responsible for gene repression through histone modification, inhibits dedifferentiation of mature somatic cells in *A. thaliana* roots. The same authors have pointed out that differentiated cells can also dedifferentiate and produce somatic embryos if AtPRC2 epigenetic repression is removed.

Mozgova et al. (2017) reinforced the idea that PRC2 constitutes a major barrier to hormone-mediated establishment of embryogenic competence in plants since lowering the PRC2-imposed barrier combined with activation treatment, the embryogenic competence is reestablished in different tissue types.

Nic-Can et al. (2015) have published an interesting demonstration on the effect of the phenolic compounds, caffeine, and chlorogenic acid in somatic embryogenesis of *C. canephora*. These authors have provided evidence that those compounds released by somatic cells to the culture medium interfere with the embryogenic process by affecting directly or indirectly DNA methylation. According to the same authors, these compounds inhibit the activity of methyltransferases. This inhibitory effect has been previously demonstrated by Lee and Zhu (2006) and by Nic-Can et al. (2013) who have produced interesting documentation on the role of epigenetic mechanisms, namely DNA methylation and histone methylation, on somatic embryogenesis competence in *C. canephora*.

Several proteins, namely B3 VP1/ABI3-LIKE (AtVAL) AtVAL1 and AtVAL2 have been reported as suppressors of somatic embryogenesis (Guan et al., 2016). They may function as repressors of AtLEC genes (Suzuki et al., 2007). According to Yang et al. (2013), At VAL proteins inhibit the expression of embryonic genes by induction of the polycomb repressive complex 1, PRC1-mediate histone H2A ubiquitination (H2Aub), and maintenance of its repression thereafter by PRC2-mediated histone H3 lysine 27 trimethylation (H3K27me3). Bouyer et al. (2011) have previously suggested the role of AtPRC1 and AtPRC2 in the transition from the embryonic to the post-embryonic stage by repression of AtLEC genes. According to Ikeuchi et al. (2015), AtPRC2 inhibits dedifferentiation of mature somatic cells in Arabidopsis roots. These authors also verified that these differentiated cells were able to dedifferentiate and give rise to somatic embryos if AtPRC2 epigenetic repression is withdrawn.

Li et al. (2014) have demonstrated that histone deacetylases (HDACs) play a negative role in induction of gametic embryogenesis in *B. napus*. This assumption resulted from the use of trichostatin A (an inhibitor of HDACs) in the culture medium that resulted in a large increase of embryogenic mass production from the *B. napus* male gametophyte.

## Final Remarks

Cell fate change is a key factor for success in plant somatic embryogenesis.

In spite of the huge progress in the obtention of somatic embryos from a myriad of plant species, including woody species, a matter of fact is that for many of them, this is still a trial-and-error process.

In woody species, in particular in forest ones, most of them recalcitrant or very difficult to produce somatic embryos, switching on the developmental program of adult cells to meristematic ones may be very time and money consuming.

Several authors namely Liu et al. (2014), Kareem et al. (2015), Efroni (2018), Rosspopoff et al. (2017), and Sang et al. (2018) have contributed a lot for the understanding of the first events related to the expression of competence for somatic embryogenesis.

A general assumption is that the regeneration process does not comprise a single step. According to those authors, several steps may occur, in particular: (1) acquisition of embryogenic competence, (2) re-specification of cell fate, and (3) cell cycle re-entry.

Much progress has been achieved in the understanding of basic processes of dedifferentiation, but a comprehensive knowledge of the epigenetic control of cell dedifferentiation is still far from being generalized and somatic embryogenesis in woody species continues to be strongly genotype dependent.

Studies on reprogramming adult cells in terms of definition of facts responsible for changes in regulation of genes will be of most interest to unveil basic mechanisms regulating cellular plasticity.

Characterization of reprogramming-related genes and proteins will provide a deeper insight into the mechanisms and networks involved in the expression of competence for embryogenesis. The control of the crucial step of fate change from differentiated to undifferentiated stage combined with the control of further steps in the somatic embryogenesis process will constitute a real opportunity to apply this technology for the improvement of forest tree species and potentially for the applicability of somatic embryogenesis in the production of synthetic seeds and its use for the benefit of reforestation programs.

Combining the information gathered on SE induction in model plants, one can expect to improve the expression of competence in woody species, making use of the molecular means available.

The capacity of deciphering the genome of woody species is growing at an incredible speed. The data generated by transcriptomics, combined with those of proteomics and metabolomics will have a tremendous impact on the knowledge of the molecular mechanisms underlying somatic embryogenesis induction. Knowledge on transcripts associated with the expression of competence for SE will allow its use as markers of competence for embryogenesis which is of most importance for achieving more accurate selection of donor explants and higher reliability of the embryogenic process in woody plants.

Deciphering genes related to signal transduction pathways like SERK (SOMATIC EMBRYOGENESIS RECEPTOR-LIKE KINASE) is of crucial importance to determine the specific conditions needed to switch on of different signal cascades during somatic embryogenesis induction.

Also, knowledge on genes encoding transcription factors such as BBM, LEC1, and LEC2 brings insights into the regulation of somatic embryogenesis induction and may have a potential to be used in somatic embryogenesis control, in particular in the expression of competence for embryogenesis.

Proteome data can also reveal markers that may constitute useful tools in the definition of conditions for induction of embryogenesis competence.

Combining data from genome, transcriptome, proteome, and metabolome may help in the identification of regulatory mechanisms and networks responsible for orchestrating the reprogramming of gene expression and may undoubtedly give insights into the dedifferentiation process giving rise to SE induction.

The roles of elements, auxins, stress, proteins, and polyamines together with their putative interaction may contribute to understand the complex interplay of signals resulting in the switch of the somatic embryogenesis process.

Data obtained from metabolome studies may help in the definition of metabolites controlling cell fate change from differentiated cells to dedifferentiated ones, a condition essential for somatic embryogenesis induction. It will also help in defining the metabolites that may function as inhibitors of somatic embryogenesis induction.

The definition of efficient embryogenic systems capable of generating high yields of somatic embryos will facilitate the use of these embryos for the obtention of large numbers of plants and its use in crop improvement (in particular woody species) making use of new breeding techniques and obtention of different lines for further screening of the desired trait(s).

## Author Contributions

The author confirms being the sole contributor of this work and has approved it for publication.

### Conflict of Interest Statement

The author declares that the research was conducted in the absence of any commercial or financial relationships that could be construed as a potential conflict of interest.
